# Surface modification of carbon nanotubes by using iron-mediated activators generated by electron transfer for atom transfer radical polymerization

**DOI:** 10.1039/c8ra00988k

**Published:** 2018-03-21

**Authors:** Yingjie Wang, Chun Tian, Hongjuan Jiang, Lifen Zhang, Xiulin Zhu

**Affiliations:** Suzhou Key Laboratory of Macromolecular Design and Precision Synthesis, Jiangsu Key Laboratory of Advanced Functional Polymer Design and Application, State and Local Joint Engineering Laboratory for Novel Functional Polymeric Materials, Department of Polymer Science and Engineering, College of Chemistry, Chemical Engineering and Materials Science, Soochow University Suzhou 215123 China zhanglifen@suda.edu.cn jianghongjuan@126.com +86-512-65882787 +86-512-65882787; Changzhou Huake Polymers Co., Ltd. No. 602 Yulong Road, Xinbei District Changzhou 213125 China; Global Institute of Soft Technology No. 5 Qingshan Road, Suzhou National Hi-Tech District Suzhou 215163 China

## Abstract

Herein, a surface-initiated activator generated by electron transfer for an atom transfer radical polymerization (AGET ATRP) system was developed on the surface of multiwall carbon nanotubes (MWCNTs) by using FeCl_3_·6H_2_O as the catalyst, tris-(3,6-dioxoheptyl) amine (TDA-1) as the ligand and ascorbic acid (AsAc) as the reducing agent. A wide range of polymers, such as polystyrene (PS), poly(methyl methacrylate) (PMMA) and poly(poly(ethylene glycol) methyl ether methacrylate) (PPEGMA), were successfully grafted onto the surfaces. The core–shell structure of MWCNTs@PS was observed by TEM. Both Raman spectra and the results of hydrolysis of MWCNTs@PS (after extraction by THF) confirmed that the PS chains were covalently tethered onto the surfaces of the MWCNTs. Due to superior biocompatibility of the iron catalyst, the strategy of modification of MWCNTs *via* iron-mediated AGET ATRP provided a promising method for the controllable and biocompatible modification of nanomaterials.

## Abstract 2

A surface-initiated AGET ATRP system was developed on the surface of multiwall carbon nanotubes by using FeCl_3_·6H_2_O as the catalyst, tris-(3,6-dioxoheptyl) amine as the ligand and ascorbic acid as the reducing agent.

## Graphical abstract

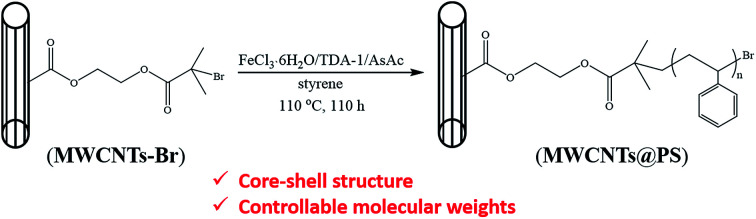

## 1. Introduction

Since carbon nanotubes (CNTs) were discovered by Iijima in 1991,^1^ it has opened up a new chapter in the development of carbon science. Their unique structure and physicochemical properties have drawn people's attention and brought us into a new era of nanotechnology.^2^ Due to their small size, high mechanical strength, large specific surface, high conductivity and strong interface effect, CNTs have special mechanical, physical and chemical properties. However, since the CNTs cannot dissolve in solvents, and are not easy to disperse in most polymers, their practical application is limited and the properties cannot be fully demonstrated. Therefore, the chemical modification of CNTs has attracted great attention from researchers.

CNTs have special specific surface area and interstitial structure. The incomplete coordination of the atoms on the surface of the CNTs leads to an increase of the active sites on the surfaces, which also provides available strategies for their surface modification. For the purpose of improving processability and expanding applications, there are many surface modification methods for CNTs, including almost all available chemical reactions.^3^ The chemical modification methods reported so far mainly involve in the introduction of carboxylic acid groups on the surface of the CNTs by carboxylic acid treatment, followed by chlorination, alcoholization or amination, thereby introducing a polymer molecular layer on the surface of the CNTs.^4^ In general, polymers can be grafted onto the surface of CNTs by both direct grafting and *in situ* grafting.^5^

So far, there have been a variety of polymers including PS, PMMA and their copolymers successfully grafted onto the surface of CNTs. Shaffer *et al.* grafted PS on multi-walled carbon nanotubes (MWCNTs) using *in situ* free radical polymerization for the first time.^6^ With anionic polymerization employed, PS and poly(*N*-vinyl carbazole) (PVK) chains were also successfully grafted onto the surface of MWCNTs. However, in order to graft polymer chains on CNTs conveniently and controllably, there is an urgent need for more efficient grafting methods. Living radical polymerization (LRP), especially atom transfer radical polymerization (ATRP) has been proven to be a good controllable method for the surface modification of solid materials. Zhu and Cheng's group carried out some works about grafting modification on various solid surfaces, including surface functionalization,^7^ synthesis of magnetic nanomaterials^8^ and so on.^9^ LRP can effectively control the molecular weight and its distribution of the grafting polymers, which grafted on the surface of solid matrix such as silicon, carbon black, Fe_3_O_4_ and other nanoparticles. Kong *et al.* used ATRP method to graft amphiphilic block copolymer brushes onto the surface of silicon.^10^ Yang *et al.* synthesized four kinds of well-defined polymers using 4-hydroxyl-2,2,6,6-tetramethylpi-peridin-1-oxyl (HTEMPO)-mediated radical polymerization.^11^ These polymers were grafted onto the surface of carbon black using radical trapping method. Dispersion experiments demonstrated that the carbon black grafted with polymers could be well dispersed in most of organic solvents. In addition, if the carbon black grafted with poly(4-vinylpyridine) was quarternized with iodomethane, it can become hydrophilic material, which has a good application prospects in sensor materials field. Wang *et al.* synthesized Fe_3_O_4_ magnetic nanoparticles (MNP) grafted with styrene and acrylic acid, using reversible addition fragmentation chain transfer (RAFT) method.^12^ Well defined polymers was obtained and characterized by gel permeation chromatography (GPC). Transmission electron microscopy (TEM) images showed that the product had a core–shell structure.

In recent years, modified CNTs has a good application on the photoelectric materials. Wei *et al.* synthesized ZnS/carbon nanotube nanocables by a two-step vapor deposition method.^13^ The product has good conductance as well as obvious light response. Vannikov *et al.* investigated the effect of cyanine dye additives on the photoelectric and photorefractive properties of polyvinyl carbazole composites based on closed single walled carbon nanotubes.^14^ In addition, the biomedical applications are also very promising.^15^ For example, Pan *et al.* utilized polyamide dendrimers modified CNTs as gene carriers and investigated the effect of dendrimer's algebra on the performance of gene vectors.^16^ Lay *et al.* put the anticancer drug paclitaxel on poly(ethylene glycol) (PEG) grafted CNTs and studied its application in treatment of cancer. They found that the delivery system can efficiently kill HeLa and MCF-7 cancer cells.^17^ Vannikova *et al.* found that the biocompatibility and low cytotoxicity of CNTs are attributed to size, dose, duration, testing systems, and surface functionalization.^18^ They functionalized CNTs to improve its solubility and biocompatibility and reduce its cytotoxic effects.

Considering the biological toxicity of copper and the superior biocompatibility of iron catalyst,^19^ we employed the iron-catalyzed AGET ATRP for the surface modification of MWCNTs, which provided a viable method for the synthesis of biomedical materials.

## 2. Experimental section

### 2.1. Materials

Poly(ethylene glycol) methyl ether methacrylate (PEGMA, 99%) was purchased from Aldrich-Sigma and passed through a neutral alumina column before use. Styrene (St, chemically pure) and methyl methacrylate (MMA, chemically pure) were purchased from Shanghai Chemical Reagents and washed with 5% NaOH three times, washed with deionized water until neutral, dried with anhydrous MgSO_4_, purified by vacuum distillation and stored in a freezer before use. Trichloromethane (CHCl_3_, analytical reagent) and trimethylamine (TEA, analytical reagent) were purchased from Shanghai Chemical Reagents and dried with molecular sieve (4 Å), purified by distillation before use. *N*,*N*-Dimethylformamide (DMF, analytical reagent), thionyl chloride (SOCl_2_, analytical reagent), ethylene glycol (99%), 4-dimethylaminopyridine (DMAP, analytical reagent), hexahydrate high ferric chloride (FeCl_3_·6H_2_O, 99%), ascorbic acid (AsAc, 99%) and nitric acid (HNO_3_, 60%) were purchased from Shanghai Chemical Reagents and used as received. Tetrahydrofuran (THF, analytical reagent), methanol were purchased from Yangyuan Chemical Factory, tris-(3,6-dioxoheptyl) amine (TDA-1, 97%) was purchased from Linhai Xinhua Chemical Factory, and multi-walled carbon nanotubes (MWCNTs) was purchased from Shenzhen Nano Harbor Co. Ltd and used as received. All other chemicals were obtained from Shanghai Chemical Reagents Co. Ltd and used as received unless mentioned.

### 2.2. Acidification of MWCNTs

5.0 g MWCNTs and 100 mL HNO_3_ (60%) were added in a 250 mL single-necked flask and it was placed in an ultrasonic bath for 30 min. Then, the reaction flask was transferred into an oil bath and heated to reflux (120 °C) with vigorous stirring. After keeping the reflux for 72 h, the mixture was cooled to room temperature and diluted with 200 mL deionized water. Then the diluted solution was filtered with a Buchner funnel and the filtrate was repeatedly washed with deionized water until the pH of the filtrate nearly neutral. 4.1 g MWCNTs-COOH was obtained after drying in a vacuum oven.

### 2.3. Synthesis of MWCNTs-OH

Added 100 mL SOCl_2_ into a 250 mL single-necked flask with 2.0 g MWCNTs-COOH as well as a magnetic stir bar and put it into an ultrasonic bath for 30 min. Then, the reaction flask was transferred into an oil bath under 65 °C and kept stirring for 48 h. The solid product was filtered and washed with dry THF. After that, the washed solid product was dried under vacuum at room temperature and 1.92 g MWCNTs-COCl was obtained.

Subsequently, in a 100 mL single-necked flask with a magnetic stir bar, 60 mL ethylene glycol and 1.2 g MWCNTs-COCl were added. After ultrasonic dispersion for 30 min, the flask was placed in a 120 °C oil bath and stirring for 48 h. The solid product was still filtered through a Buchner funnel, and washed repeatedly with THF. 1.11 g MWCNTs-OH was obtained after dried under vacuum at room temperature.

### 2.4. Synthesis of MWCNTs-Br initiator

1.0 g MWCNTs-OH, 30 mL dry CHCl_3_, 0.073 g DMAP and 0.76 g dry TEA were added into a 100 mL three-necked flask with a magnetic stir bar and the mixture was placed in an ultrasonic bath for 30 min. Slowly added acyl bromide solution (0.96 g 2-bromoisobutyryl bromide and 13 mL dry CHCl_3_) into the flask under ice bath and Ar atmosphere for about 1 h. Then the mixture was stirred at 0 °C for 3 h and then at room temperature for 48 h. The solid product was filtered with a Buchner funnel, and washed repeatedly with CHCl_3_. After that, the initial product was dissolved in 30 mL CHCl_3_ and placed in an ultrasonic bath and filtered. Washed repeatedly with CHCl_3_ until no residual 2-bromoisobutyryl bromide. 0.94 g MWCNTs-Br initiator was obtained after dried under vacuum.

### 2.5. Typical procedure for the surface-initiated AGET ATRP on MWNCTs

A typical polymerization procedure with the molar ratio of [St]_0_ : [FeCl_3_·6H_2_O]_0_ : [TDA-1]_0_ : [AsAc]_0_ = 50 : 1 : 3 : 1 and mass ratio of St/MWCNTs-Br = 25 : 1 was conducted as follows. In a dried 5 mL ampule with a magnetic stir bar, MWCNTs-Br (25.0 mg), FeCl_3_·6H_2_O (13.0 mg), TDA-1 (46.6 mg) and DMF (1.0 mL) were added. The mixture was placed in an ultrasonic bath for 15 min. After that, St (0.275 mL) and AsAc (8.4 mg) were added in the mixture and then the ampule was bubbled thoroughly with Ar for 20 min to eliminate the dissolved oxygen in the reaction system and flame-sealed. The ampule was transferred into an oil bath keeping it at 110 °C. After the desired polymerization time, the ampule was diluted with 10 mL of CHCl_3_, precipitated with methanol and filtered. The product was dried under vacuum to a constant weight. In order to remove the homopolymer from the product, it was extracted with THF using a Soxhlet extractor for 72 h. The final product was redispersed with 5 mL CHCl_3_, precipitated with methanol and filtered, and MWCNTs@PS were obtained after vacuum drying. The polymerization procedures of other monomers were the same as mentioned above.

### 2.6. Hydrolysis of MWCNTs@PS

In order to obtain the grafting polymers for GPC analysis, 40 mg MWCNTs@PS were dispersed in 40 mL THF and then adding 10 mL 1 M KOH/ethanol solution and refluxing for 72 h with stirring. The mixture was centrifuged at 1500 rpm for 10 min to obtain de-functionalized MWCNTs (bottom of centrifuge tube) and PS dissolved in THF (supernatant) hydrolyzed from MWCNT@PS. The supernatant was precipitated with a large amount of methanol (∼500 mL), let stand overnight and then filtered with a Buchner funnel. After that, grafting PS hydrolyzed from MWCNTs@PS were obtained after vacuum drying.

### 2.7. Characterization

^1^H NMR spectra were recorded on an INOVA 400 MHz nuclear magnetic resonance spectrometer using CDCl_3_ as a solvent and tetramethylsilane (TMS) as an internal standard. Transmission Electron Microscopy (TEM) was performed using TecnaiG220 with an acceleration voltage of 200 kV. Infrared spectroscopic analysis was measured by a KBr pellet using Nicolet 1300. Thermal Analysis (TGA) using the SDT 2960 and the heating rate was 10 °C min^−1^ under N_2_ atmosphere. Raman spectroscopy was recorded by HR800. The molecular weight (*M*_n,GPC_) and molecular weight distribution (*M*_w_/*M*_n_) of the resultant polymers were determined using a Waters 1515 gel permeation chromatography (GPC) equipped with refractive index detector (Waters 2414), using HR1, HR2 and HR3 columns (7.8 × 300 mm) with measurable molecular weights ranging from 10^2^ to 5 × 10^5^ g mol^−1^. THF was employed as the eluent at a flow rate of 1.0 mL min^−1^ and 30 °C. GPC samples were injected using a Waters 717 plus autosampler. The grafting PS molecular weights were calibrated with PS standards and grafting PMMA were calibrated with PMMA standards, respectively.

## 3. Results and discussion

### 3.1. Immobilization of initiator MWCNTs-Br

**Scheme 1:**
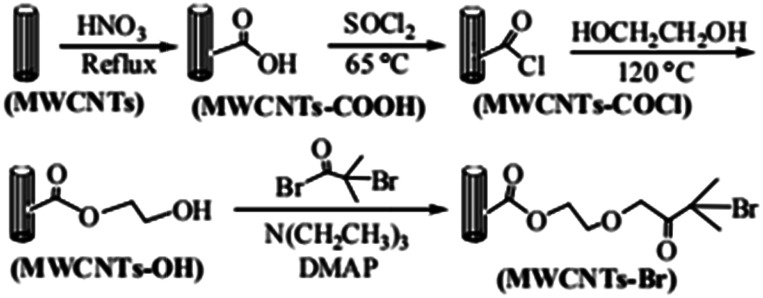
Schematic diagram illustrating the process for the immobilization of ATRP initiators on MWCNTs.

The route of immobilization of initiator MWCNTs-Br is shown in Scheme 1. Firstly, carboxyl groups are attached to the surface of MWCNTs by acidification with nitric acid. Secondly, carboxyl groups reacts with thionyl chloride and converted to acid chloride groups and then converted to hydroxy esters by reaction with ethylene glycol. Finally, after the reaction with acyl bromide, initiating group 2-bromoisobutyrate is immobilized on the surface of the MWCNTs. In our experiments, the filtration and washing procedure is necessary in each step. Small molecules adsorbed on MWCNTs must be completely removed to ensure the purity of MWCNTs-Br.

**Fig. 1:**
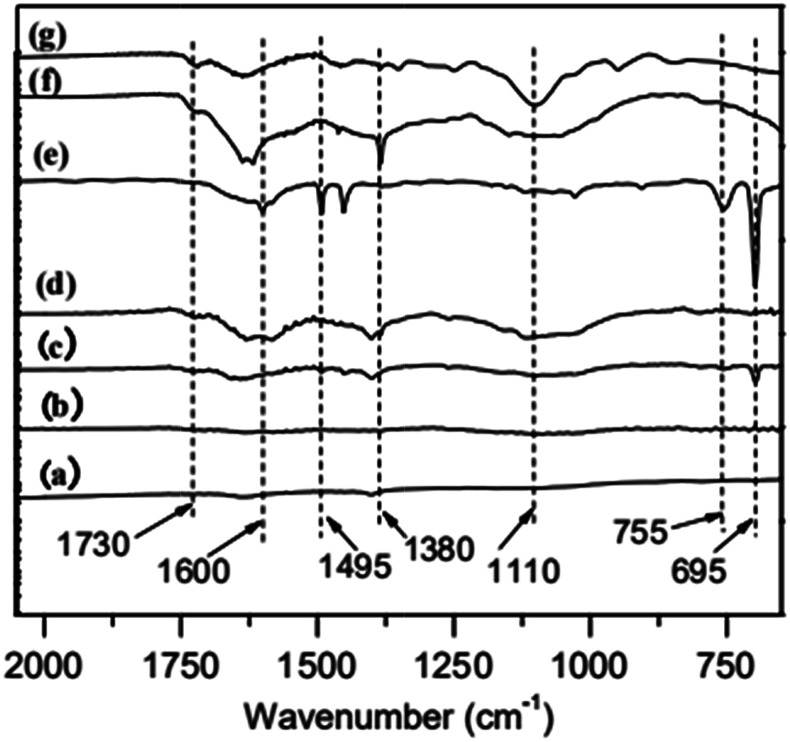
FT-IR spectra of (a) original MWCNTs, (b) MWCNTs-COOH, (c) MWCNTs-OH, (d) MWCNTs-Br, (e) MWCNTs@PS, (f) MWCNTs@PMMA and (g) MWCNTs@P(PEGMA).

Fourier transform infrared (FT-IR) spectroscopy is used to characterize the immobilization of ATRP initiator on MWCNTs. The results are shown in Fig. 1a–d. MWCNTs-OH (c), a product of ethylene glycol grafting on MWCNTs, has an obvious C=O peak at 1730 cm^−1^. As for MWNCTs-Br (d), the product of MWCNTs-OH reacts with 2-bromoisobutyryl bromide, its intensity of C=O peak is increased, which indicates the initiator was immobilized on the surface of MWCNTs.

**Fig. 2:**
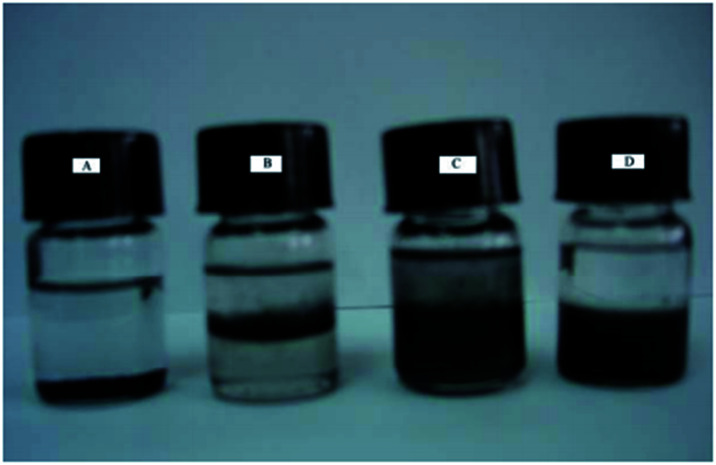
Photos of original MWCNTs (A) dispersed in CHCl_3_, MWCNTs-COOH (B), MWCNTs-OH (C) and MWCNTs-Br (D) dispersed in CHCl_3_/H_2_O mixture.

In the synthesis of MWCNTs-Br, MWCNTs dispersion has undergone great changes. As is shown in Fig. 2, MWCNTs cannot be well dispersed in any solvents we used (Fig. 2A). After acidification, the MWCNTs-COOH can partially disperse in water and gathered at the interface between CHCl_3_ and H_2_O (Fig. 2B). MWCNTs-OH showed better dispersibility than MWCNTs-COOH in water and organic solvents (Fig. 2C). However, MWCNTs-Br shows very poor dispersibility in water, and relatively good dispersibility in organic solvents (Fig. 2D). This is mainly due to the good hydrophilicity of hydroxyl and carboxyl groups on the surface of MWCNTs while 2-bromo isobutyrate has poor hydrophilicity.

### 3.2. Surface-initiated AGET ATRP

To illustrate the versatility of iron-catalyzed AGET ATRP in surface modification, we select three typical monomers, St, MMA and PEGMA, which were initiated on the surfaces of MWCNTs-Br. In order to remove the homopolymer in the product, all samples were extracted by THF. As is shown in Fig. 1e–g, the characteristic peaks in PS (695 cm^−1^, 755 cm^−1^, 1495 cm^−1^ and 1600 cm^−1^),^20^ PMMA (1380 cm^−1^ and 1730 cm^−1^)^21^ and P(PEGMA) (1100 cm^−1^ and 1730 cm^−1^)^22^ are obvious, which means grafting polymers MWCNTs@PS, MWCNTs@PMMA and MWCNTs@P(PEGMA) are obtained.

In addition, considering that the methacrylates polymers cannot be perfectly hydrolyzed from the MWCNTs, we selected MWCNTs@PS as the grafting polymers for the characterization of molecular weights; namely, the grafting PS polymers were obtained by hydrolysis of MWCNTs@PS. The polymerization conditions and the results are shown in Table 1. As the weight ratio (*R*_1_) of styrene to MWCNTs-Br gradually increases, the number average molecular weight of grafted PS (*M*_n,GPC_) increases, indicating the molecular weight of PS grafted onto the surface of MWCNTs can be controlled by adjusting the feed ratio of monomer to initiator *via* iron-mediated AGET ATRP. At the same time, the resultant molecular weight distribution of grafting PS is relatively broad (*M*_w_/*M*_n_ ∼ 2.0) but narrower than that reported in the literatures (*M*_w_/*M*_n_ ∼ 3.0).^3^ The broad *M*_w_/*M*_n_ values may be due to the following facts: MWCNTs are not uniform and even small amount of single-walled carbon nanotubes may exist, resulting in different surface conditions of MWCNTs. Even on the same MWCNTs, the wider molecular weight distribution can be caused by the different density of immobilized initiators due to different distributions of defects and different degrees of oxidation.

**Table 1:** Molecular weight and molecular weight distribution of the grafting PS^a^

Sample	*R*_1_	*R*_2_	Time (h)	*M*_n,GPC_ (g mol^−1^)	*M*_w_/*M*_n_
MWCNTs@PS-1	10/1	50/1/3/1	110	3400	1.94
MWCNTs@PS-2	25/1	50/1/3/1	110	46 800	1.93
MWCNTs@PS-3	50/1	50/1/3/1	110	84 100	2.13

a *R*_1_ = St/MWCNTs-Br (w/w); *R*_2_ = [St]_0_/[FeCl_3_·6H_2_O]_0_/[TDA-1]_0_/[AsAc]_0_.

### 3.3. TGA and ^1^H NMR characterization

**Fig. 3:**
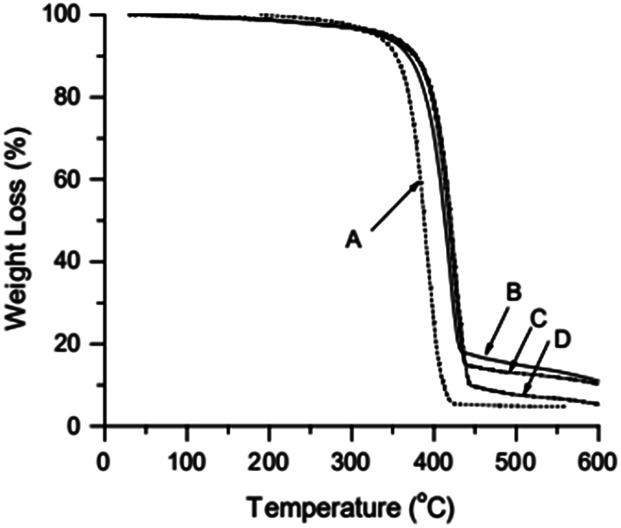
TGA curves of PS (A), MWCNTs@PS-1 (B), MWCNTs@PS-2 (C) and MWCNTs@PS-3 (D).

TGA curve for MWCNTs@PS and homopolymer PS is shown in Fig. 3. The PS decomposition temperature (*T*_d_) of MWCNTs@PS is close to 390–400 °C, about 40–50 °C higher than homopolymer PS (350 °C). This is due to the synergistric effect of MWCNTs, which has high thermal stability, and the grafting PS chains. It is noted that from the residual weight% of the MWCNTs@PS the functionality of MWCNTs seemed not much high, indicating that the functionalization has happened but not quantitatively and efficiently.

**Fig. 4:**
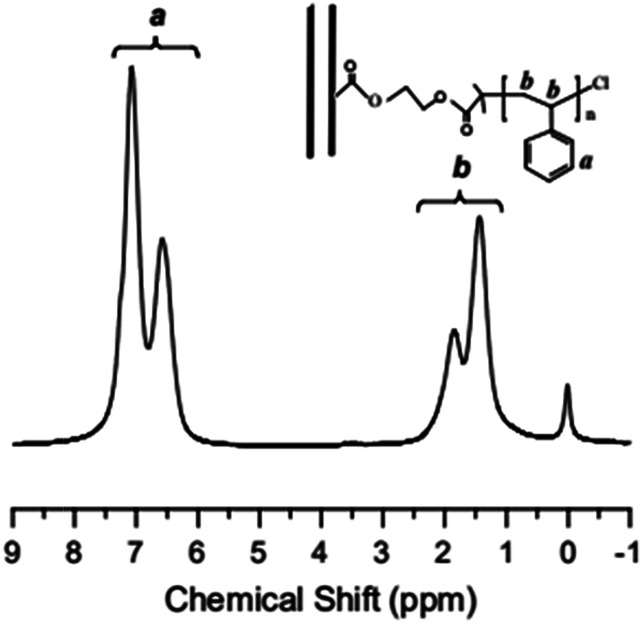
^1^H NMR spectrum of MWCNTs@PS-2 (*M*_n,GPC_ = 46 800 g mol^−1^, *M*_w_/*M*_n_ = 1.93) obtained by AGET ATRP with DMF as the solvent.

Fig. 4 shows the ^1^H NMR spectrum of MWCNTs@PS-2. It is obvious that the chemical shift at 6.5–7.2 ppm ((a) in Fig. 4) corresponds to the characteristic peak of benzene ring and the chemical shift at 1.0–2.0 ppm ((b) in Fig. 4) belongs to methylene and methine groups. According to the literature, electron absorption effect of chlorine atom at PS chain end will lead to a chemical shift of methine of PS chain end at 4.2–4.5 ppm.^23^ When the molecular weight of PS is too high, the unique signal peaks become too weak to be easily observed. However, the presence of these peaks above demonstrates that PS is indeed grafted onto the MWCNTs.

### 3.4. Raman and TEM characterization

**Fig. 5:**
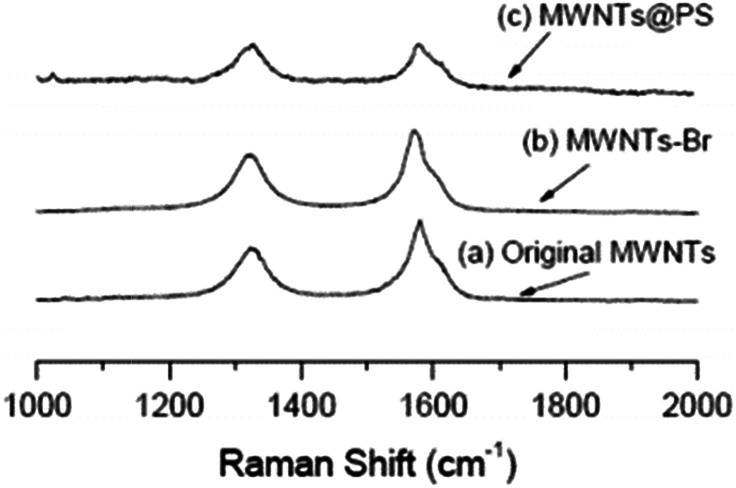
Raman spectra of original MWCNTs (a), MWCNTs-Br (b) and MWCNTs@PS (c). The laser wavelength is 632.8 nm and the laser power used is 6 mW.

It is well known that Raman spectroscopy can be used to characterize the presence and proportion of D-line (amorphous carbon and disordered induction line) and G-line in MWNTs. The Raman spectra of MWCNTs, MWCNTs-Br and MWCNTs@PS are shown in Fig. 5. Two peaks at 1320 cm^−1^ and 1580 cm^−1^ respectively correspond to the characteristic peaks of the D-line and G-line in MWCNTs.^24^ At the same time, according to the intensity of these two peaks ratio (*I*_D_/*I*_G_), the ratio of the D-line in MWCNTs, MWCNTs-Br and MWCNTs@PS is 0.69, 0.84 and 0.97 respectively. ATRP initiator fragments and PS grafts are linked by covalent bonds with MWNTs, leading to the transformation of sp^2^ hybridized carbon atom to sp^3^ hybridized carbon atom in original MWCNTs and a higher *I*_D_/*I*_G_ value.

**Fig. 6:**
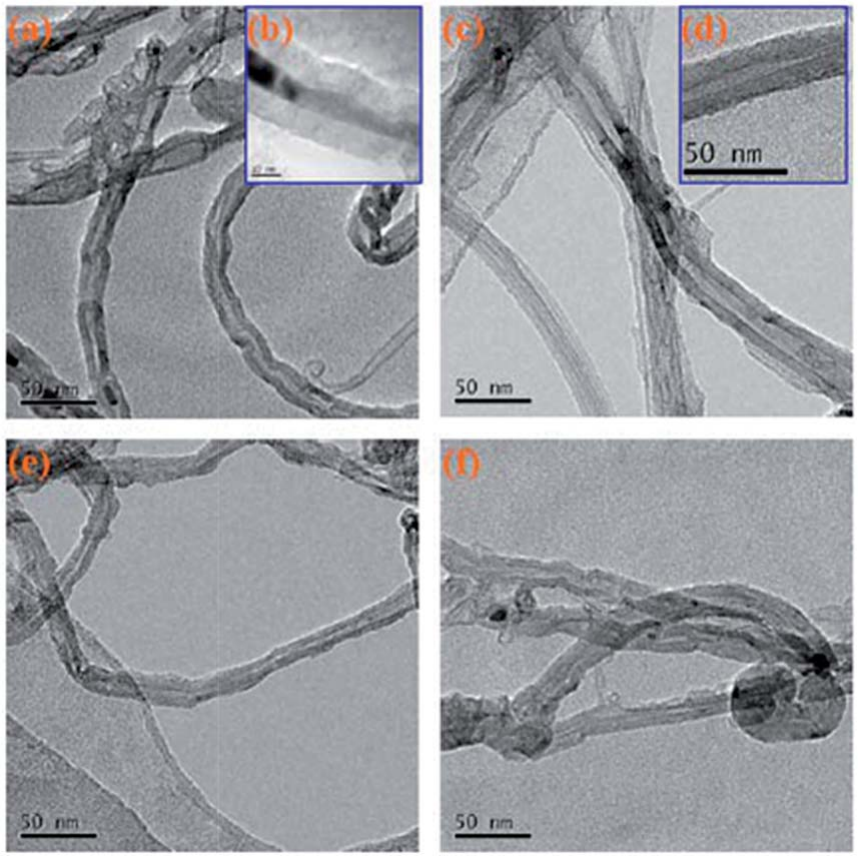
TEM images of original MWCNTs (a, b), MWCNTs@PS (c, d), MWCNTs@PMMA (e) and MWCNTs@P(P(PEGMA) (f).

Fig. 6 shows a TEM image of the original MWCNTs and the modified MWCNTs. As can be clearly seen from Fig. 6a, the MWCNTs is not uniform in size and there is also a portion of amorphous carbon nanotube impurities present. Fig. 6c and d show the TEM images of MWCNTs@PS. We can clearly see that the outer wall of the MWCNTs is covered with a layer of polymer. Fig. 6e and f also show a core–shell structure, which means the corresponding polymers were successfully grafted onto MWCNTs.

### 3.5. X-ray photoelectron spectroscopy (XPS) characterization

**Fig. 7:**
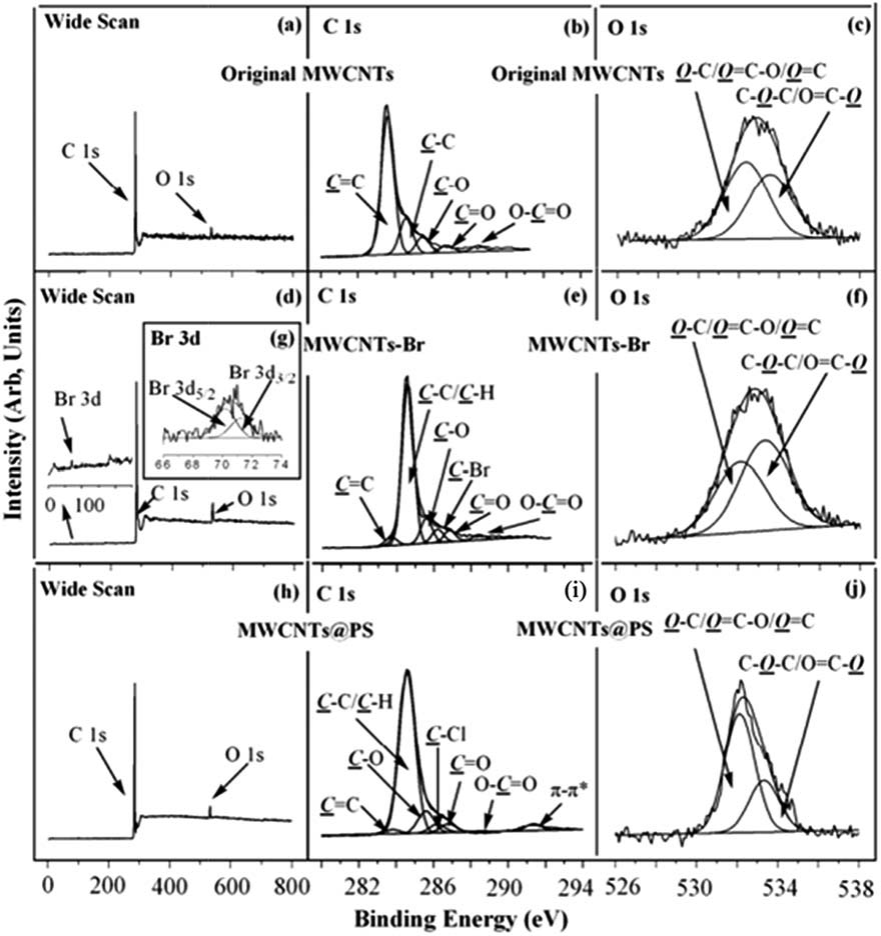
XPS (a) wide scan, (b) C 1s, and (c) O 1s core-level spectra of the original MWCNTs; XPS (d) wide scan, (e) C 1s, (f) O 1s, and (g) Br 3d (inset in the wide scan spectrum) core-level spectra of the MWCNTs-Br; XPS (h) wide scan, (i) C 1s, and (j) O 1s core-level spectra of the resultant MWCNTs@PS.

XPS is one of the most powerful tools for characterizing chemical structures and chemical compositions of solid surfaces. XPS wide scan, C 1s, and O 1s core-level spectra of the original MWCNTs are shown in Fig. 7a–c. The characteristic peaks of C 1s and O 1s correspond to 284 eV and 533 eV of XPS wide scan spectrum of the original MWCNTs (Fig. 7a).^25^ Binding energy at 283.6, 284.6, 285.5, 286.8 and 288.5 eV belong to the C=C, C–C, C–O, C=O and O=C–O characteristic peaks (Fig. 7b), which correspond to sp^2^ hybrid carbon atoms; sp^3^ hybrid carbon atoms; alcohol, ether structure; carbonyl and carboxyl in the original MWCNTs respectively.^26^ Binding energy at 532.4 and 533.6 eV correspond to the characteristic peaks of HO–C/O=C–O/O=C and O=C–O/C–O–C in MWCNTs (Fig. 7c).^25^

Fig. 7d–g show the XPS wide scan, C 1s, and O 1s core-level spectra of MWCNTs-Br. Binding energy at 71 eV, 284 eV and 533 eV correspond to the characteristic peaks of Br 3d, C 1s and O 1s of MWCNTs-Br (Fig. 7d).^25^ The appearance of a new Br 3d peak in the wide scan spectrum as well as binding energy at 70.2 eV and 71.3 eV in the Br 3d core-level spectra (Fig. 7g) indicate that the initiator 2-bromoisobutyrate has been successfully immobilized on the surface of MWCNTs. Binding energy at 286.2 eV corresponds to the covalently linked C–Br characteristic peak,^25^ which further demonstrates the successful immobilization of the initiator (Fig. 7e). Moreover, the five characteristic peaks (283.6, 284.9, 285.6, 286.9 and 288.4 eV respectively correspond to C=C, C–C, C–O, C=O and O=C–O) of original MWCNTs still exist, which indicates that the thickness of the initiator layer is less than the XPS detection depth (about 7.5 nm (ref. 27)). This is consistent with the fact that organic chemical reactions on solid surfaces often generate monomolecular layers.

XPS wide scan, C 1s, and O 1s core-level spectra of MWCNTs@PS are shown in Fig. 7h–j. There is a strong new peak at 291.4 eV (Fig. 7i) belongs to π–π conjugated characteristic peak of benzene ring,^25^ which demonstrates that PS successfully grafted on the surface of MWCNTs. In addition, since there is no oxygen atom in PS, if the surface of entire MWCNTs is covered by PS layer, a signal of O 1s should not be detected. However, the existence of O 1s signal (Fig. 7h and j) and the five characteristic peaks (Fig. 7i, 283.6, 284.9, 285.6, 286.9 and 288.4 eV respectively correspond to C=C, C–C, C–O, C=O and O=C–O) prove that the thickness of the PS grafted onto the MWCNTs is less than XPS detection depth (7.5 nm (ref. 27)).

## 4. Conclusions

In summary, ATRP initiator was successfully immobilized on MWCNTs by a 4-step method, and different kinds of polymers were successfully grafted by iron-catalyzed surface-initiated AGET ATRP. The core–shell structure of MWCNTs@PS was proved by TEM. The MWCNT@PS hydrolysis defunctionalization after THF extraction and Raman spectroscopy both demonstrated that the modified MWCNTs and PS were linked by covalent bonds. Therefore, a promising method for the controllable, facile and biocompatible surface modification of nanomaterials was established.

## Conflicts of interest

There are no conflicts to declare.

## Supplementary Material

## References

[cit1] Iijima S. (1991). Nature.

[cit2] Chen J., Hamon M. A., Hu H., Chen Y., Rao A. M., Eklund P. C., Haddon R. C. (1998). Science.

[cit3] Lee J., Jun J., Cho S., Kim W., Jang J. (2017). RSC Adv..

[cit4] Pei X., Xia Y., Liu W., Hao J. (2008). J. Polym. Sci., Part A: Polym. Chem..

[cit5] Hao K., Gao C., Yan D. (2004). J. Mater. Chem..

[cit6] Shaffer M., Koziol K. (2002). Chem. Commun..

[cit7] Tang F., Zhang L., Zhu J., Cheng Z., Zhu X. (2009). Ind. Eng. Chem. Res..

[cit8] Liu X., Chen Q., Yang G., Zhang L., Liu Z., Cheng Z., Zhu X. (2015). J. Mater. Chem. B.

[cit9] Li Q., Zhang L., Bai L., Zhou N., Cheng Z., Zhu X. (2011). Soft Matter.

[cit10] Kong X., Kawai T., Jiro A., lyoda T. (2001). Macromolecules.

[cit11] Yang Q., Wang L., Xiang W., Zhou J., Li J. (2007). Polymer.

[cit12] Wang W., Neoh K., Kang E. (2006). Macromol. Rapid Commun..

[cit13] Wei D., Liu Y., Cao L., Zhang H., Huang L., Yu G. (2010). Chem. Mater..

[cit14] Vannikov A., Grishina A., Laryushkin A., Krivenko T., Savel'Ev V., Rychwaiski R. (2013). Phys. Solid State.

[cit15] Campidelli S., Klumpp C., Bianco A., Guldi D., Prato M. (2006). J. Phys. Org. Chem..

[cit16] Pan B., Cui D., Xu P., Ozkan C., Feng G., Ozkan M. (2009). Nanotechnology.

[cit17] Lay C., Liu H., Tan H., Liu Y. (2010). Nanotechnology.

[cit18] Vardharajula S., Ali S., Tiwari P., Eroğlu E., Vig K., Dennis V. (2012). Int. J. Nanomed..

[cit19] Zhang L., Cheng Z., Shi A., Li Q., Zhu X. (2008). Polymer.

[cit20] Li H., Cheng F., Duft A. M., Adronov A. (2005). J. Am. Chem. Soc..

[cit21] Chen H., Deng C., Zhang X. (2010). Angew. Chem..

[cit22] Cho W., Hong J., Lee J., Kim So., Kim Se., Im S. (2016). RSC Adv..

[cit23] Cheng Z., Zhu X., Zhou N., Zhu J., Zhang Z. (2005). Radiat. Phys. Chem..

[cit24] Rao A., Jorio A., Pimenta M., Dantas M., Saito R., Dresselhaus G. (2000). Phys. Rev. Lett..

[cit25] Davies M. (1992). Biomaterials.

[cit26] Chen C., Liang B., Ogino A., Wang X., Nagatsu M. (2009). J. Phys. Chem..

[cit27] Watts J. (1998). Surface Engineering.

